# DATMA: Distributed AuTomatic Metagenomic Assembly and annotation framework

**DOI:** 10.7717/peerj.9762

**Published:** 2020-09-03

**Authors:** Andres Benavides, Friman Sanchez, Juan F. Alzate, Felipe Cabarcas

**Affiliations:** 1 Grupo GICEI, Facultad de Ingeniería Electrónica, Institución Universitaria Pascual Bravo, Medellín, Antioquia, Colombia; 2 Barcelona Supercomputing Center, currently at Smart Variable S.L., Barcelona, Spain; 3 Centro Nacional de Secuenciación Genómica-CNSG, Sede de Investigación Universitaria-SIU, Universidad de Antioquia UdeA, Medellín, Colombia; 4 Departamento de Microbiología y Parasitología, Facultad de Medicina, Universidad de Antioquia UdeA, Medellín, Colombia; 5 Grupo SISTEMIC, Ingeniería Electrónica, Facultad de Ingeniería, Universidad de Antioquia UdeA, Medellín, Colombia

**Keywords:** Distributed computing, Bioinformatics, Grid computing, Algorithm, Workflow, Metagenomics, Workflow

## Abstract

**Background:**

A prime objective in metagenomics is to classify DNA sequence fragments into taxonomic units. It usually requires several stages: read’s quality control, de novo assembly, contig annotation, gene prediction, etc. These stages need very efficient programs because of the number of reads from the projects. Furthermore, the complexity of metagenomes requires efficient and automatic tools that orchestrate the different stages.

**Method:**

DATMA is a pipeline for fast metagenomic analysis that orchestrates the following: sequencing quality control, 16S rRNA-identification, reads binning, de novo assembly and evaluation, gene prediction, and taxonomic annotation. Its distributed computing model can use multiple computing resources to reduce the analysis time.

**Results:**

We used a controlled experiment to show DATMA functionality. Two pre-annotated metagenomes to compare its accuracy and speed against other metagenomic frameworks. Then, with DATMA we recovered a draft genome of a novel Anaerolineaceae from a biosolid metagenome.

**Conclusions:**

DATMA is a bioinformatics tool that automatically analyzes complex metagenomes. It is faster than similar tools and, in some cases, it can extract genomes that the other tools do not. DATMA is freely available at https://github.com/andvides/DATMA.

## Introduction

The analysis of metagenomic experiments, from next-generation sequencing, requires several stages: bases quality control, reads binning (optional), reads assemble, and taxonomic classification. Tools like Trimmomatic (Bolger, Lohse & Usadel, 2014), SolexaQA (Cox, Peterson & Biggs, 2010) (quality control tools), Velvet (Zerbino & Birney, 2008), MetaVelvet (Namiki et al., 2012), SPAdes (Nurk et al., 2013), metaSPAdes (Nurk, Meleshko & Korobeynikov, 2017), (assembly tools), CLARK (Ounit et al., 2015), Kaiju (Menzel, Ng & Krogh, 2016) (annotation tools), Prodigal (Hyatt et al., 2010), GeneMark (Besemer & Borodovsky, 2005) (gene prediction tools), among others, can be used to address these tasks. Many of them have been integrated into full pipelines like MetAMOS (Treangen et al., 2013), RAST server (MG-RAST) (Wilke et al., 2016), IMG/M server (Chen et al., 2017), MetaWRAP (Uritskiy, DiRuggiero & Taylor, 2018), SqueezeMeta (Tamames & Puente-Sánchez, 2019), MetaMeta (Piro, Matschkowski & Renard, 2017), MOCAT2 (Kultima et al., 2016). These pipelines allow for the processing of metagenomic datasets automatically. But currently, there is not a standard tool designed to study a metagenomic’s dataset. The design of accurate algorithms and tools is an open field of research.

Assembly is the main challenge of metagenomic analysis. Microbial communities are complex. The Bacteria of the communities have different genome sizes and abundances. Furthermore, some regions of their genome are very similar. Therefore, the sequencing of these communities results in a complex mixture of reads from the microorganisms. Chimeric molecules are still one of the main problems of de novo metagenomic assembly (Tamames & Puente-Sánchez, 2019). Despite the development of many specialized de novo assemblers for metagenomics (e.g., MetaVelvet (Namiki et al., 2012) and metaSPAdes (Nurk, Meleshko & Korobeynikov, 2017)), it is not possible to eliminate the probability of creating chimeric contigs. Moreover, most metagenome analysis pipelines start by assembling the complete read dataset. However, the vast amount of information on DNA provided by next-generation sequencing makes that this task can exceed the computing capacities. Grouping very similar reads, before assembling them, can address many of these problems. By creating bins of reads mostly from a single molecule, the assembler does not have to assemble the whole dataset and, therefore, can assemble the complete metagenome in parts.

CLAME (Benavides et al., 2018) allowed researchers to extract a nearly complete bacterial genome from a complex metagenome. However, it requires many manual steps, making it hard to use, especially with large data sets. DATMA integrates CLAME into a distributed workflow for metagenomic analysis. DATMA automatically executes: (i) sequencing quality control (ii) 16S rRNA gene sequence detection, (iii) CLAME binning, (iv) de novo assembly and contigs evaluation (v) ORF detection and taxonomic analysis, and (vi) data management report.

We designed DATMA using a distributed programming model called COMP Superscalar (COMPSs) (Badia et al., 2015). It allows DATMA to run in parallel on several threads or different computing infrastructures. COMPSs automatically exploits the application parallelism without the need of dealing with data partitioning and task distribution on the available computers. Commonly, software users and programmers manually deal with these two challenges. We show that COMPSs allows DATMA to be faster than other pipelines with similar results.

In this article we introduce DATMA. First, we describe its components and structure. Then, we evaluate its performance using controlled experiments. Finally, we test DATMA with a novel metagenome from a wastewater treatment plant. We show that DATMA extracted a novel Anaerolineaceae draft genome from this metagenome.

## Materials & Methods

**Figure 1:**
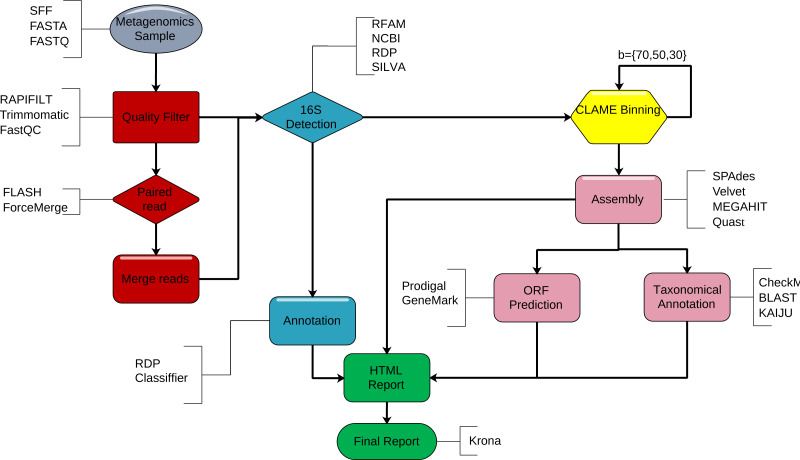
DATMA structure. DATMA automatically executes. (i) sequencing quality control (red blocks) (ii) 16S-identification (blue blocks), (iii) CLAME binning (yellow blocks), (iv) de novo assembly, ORF detection, taxonomic analysis (violet blocks) and (vi) data management report (green blocks).

DATMA is a command line software for Unix-based systems. We show DATMA’s structure in Fig. 1 and describe each stage in the following subsections.

### DATMA stages

#### Reads quality trimming and filtering

DATMA receives FASTQ, FASTA, or Standard Flowgram Files (SFF). For reads’ quality control, it uses Trimmomatic (Bolger, Lohse & Usadel, 2014) or RAPIFILT, which is a custom tool. This stage trims low-quality bases at both ends of the reads and removes the ones that are too short from the dataset. Afterwards, it uses FastQC (Andrews, 2019) to plot the quality statistics.

For pair-end reads, DATMA uses FLASH2 (Magoč & Salzberg, 2011) to extend the reads and merge them into a single (FASTA or FASTQ) file, before passing them to the next stage. If the fragment length is too large to be combined, we force the merging by adding three extra N characters between the end of the first read and the beginning of the second one, which is in reverse-complement (e.g., ATCGT  NNN TTATC). DATMA extends the reads only for the binning stage, which produces a list of reads for each bin. After binning, DATMA uses the original reads (selected in the binning) for assembly.

#### 16S rRNA genes sequences detection

In a metagenome dataset, ribosomal sequences can be used to profile the bacteria species in the sample and estimate their abundance. DATMA uses the BWA tool (Li & Durbin, 2009) to map the raw reads against a ribosomal database and remove ribosomal sequences from the pool of reads to improve the binning. This process reduces the probability that these conserved regions connect reads from different species on the same bin. DATMA aligns the reads to a reference 16S rRNA gene-database, the user can select any of NCBI-16S rRNA database (National Center for Biotechnology Information, 2019), RDP (Cole et al., 2014), Greengenes (DeSantis et al., 2006), Rfam (Griffiths-Jones et al., 2003), RNAmmer (Lagesen et al., 2007) or SILVA (Quast et al., 2013) (Table S1, in Additional File 1, details each one of them). Finally, the detected sequences are classified using the RPD-tool classifier (Wang et al., 2007).

#### CLAME binning

CLAME (Benavides et al., 2018) bins DNA sequences into groups of reads from the same molecule. It creates a graph representation of the metagenome in which reads are nodes, and their alignments are edges between them. CLAME aligns all reads against each other to create the graph. To generate the bins, the users must set the number-of-bases threshold (b-parameter), which indicates the minimum alignment size accepted. Given that the number of edges (alignments) of a node (read) is related with the abundance of that part of the molecule on the metagenome, CLAME creates the number-of-edges-per-node histogram of each bin. So the users can decide the thresholds on the number of edges (e-parameter).

DATMA uses the median absolute deviation (MAD) statistic (Leys et al., 2013) to determine the e-parameter automatically. CLAME’s authors suggest that in a bin from a single molecule, the edges histogram should have a normal-like distribution. If we assume the departure from this distribution is due to the noise produced by the similarity of regions of the genome with other genomes or repetitive zones, it is possible to use MAD to detect these reads with extreme values (outliers) and remove them.

The MAD statistic is a robust nonparametric spread measure. It is the median of the absolute deviations from the median (see eq2.1 in Additional File 2). Moreover, for a normal distribution, the MAD can be used as a consistent estimator of the population standard deviation, with SIGMA = b*MAD, with *b* = 1.4826 (see eq2.2 in Additional File 2). Then, DATMA marks reads with distance greater than 3 MAD from the median (see eq2.3 in Additional File 2) as outliers and removes them; the other reads are kept and reported in the bin. Additional File 2 and CLAME paper (Benavides et al., 2018) gives a complete description of this process.

DATMA, by default, starts with 70 (bp) as CLAME’s b-parameter. Then, it iterates with other values (e.g., using 50 bp or 30 bp) to explore the metagenome in detail. It is important to highlight that lowering the b-value increases the probability of reads from different molecules reported on the same bin. The user can modify the b-parameter using the configuration file (see DATMA’s user manual available in DATMA’s GitHub).

#### Assembly and contigs’ evaluation

DATMA assembles (de novo) all bins produced by CLAME. The user can select among different assembly tools: Velvet (Zerbino & Birney, 2008), SPAdes (Nurk et al., 2013), or MEGAHIT (Li et al., 2015). After assembly Quast tool (Gurevich et al., 2013) evaluates the contigs and report their metrics. Finally, DATMA uses CheckM program (Parks et al., 2015) to assess the quality and contamination of the bins.

#### ORF detection and taxonomic analysis

DATMA uses the assembled contigs to predict protein-coding-genes; the user can select between Prodigal (Hyatt et al., 2010) or GeneMark (Besemer & Borodovsky, 2005) for this task. Next, the contigs are annotated using BLAST (Altschul et al., 1990) and a local NT-database. DATMA also provides the Kaiju tool (Menzel, Ng & Krogh, 2016) for sensitive taxonomic classification.

#### Final report

DATMA reports the statistics of each workflow stage into an HTML file. It uses Krona (Ondov, Bergman & Phillippy, 2011) to represent the taxonomic classification into an interactive plot. Using the Krona report, the user can explore each bin classification at different taxonomic ranks and select between individual annotation of each bin or combine data from all bins. Figs. S1, S2 and S3, show an example of the output file generated by DATMA.

### Workflow design

DATMA is a command line application written in Python and tested in Linux. We provide an installation script in our GitHub to automatically install DATMA source codes and the tools that make up part of the workflow. We tested it on Ubuntu 16.04 and included a user manual for custom compilation and installation of source codes on other Linux distributions. By default, DATMA configures all tools called in the workflow according to the authors recommended parameters, but these values can be modified using a configuration file. In this file, the user specifies the input sequence file, the output directory, the workflow stages, the database directories, the number of threads to use, CLAMEs parameters, etc. The minimum configuration file should contain the input-sequence file, the sequence type (i.e., FASTA, FASTQ, or SFF) and the output directory. We show a complete configuration file in DATMA’s user manual.

Although there are several workflow engines (e.g., Snakemake (Koster & Rahmann, 2012), Nextflow (Di Tommaso et al., 2017), Ibis (Bal et al., 2010), and Swift (Wilde et al., 2011)) we selected COMPSs (Badia et al., 2015) which provided us with the tools that we required: simple python interface and automatic parallel task distribution and synchronization. COMPSs offers a simple programming model, that does not require the use of APIs to modify the original user applications, and enables the execution of the same code on different back-ends. It uses a sequential description of the work, and it identifies and launches asynchronous parallel tasks automatically. A complete description of COMPs and its performance is in Badia et al. (2015).

COMPSs allows DATMA to be executed in single or distributed mode. In single mode, the framework executes all the stages into the same computer. In distributed mode, DATMA uses a master-worker execution strategy, to distribute application tasks across the different computer nodes available. It executes the quality control, 16S rRNA identification, and CLAME binning stages in the master node (these stages can be multi-threaded). Once the bins are generated, DATMA assembles and annotates them using the available nodes. It requires two configuration files (resources.xml and project.xml) within the execution environment. The first file contains the information of the available computing resources, and the second file has information about the computing resources to be used for a specific execution. The user manual has an example of each file.

## Results

### CAMI dataset

We used the first Critical Assessment of Metagenome Interpretation (CAMI) (Sczyrba et al., 2017) challenges to evaluate DATMA performance. CAMI consortium provides three metagenome datasets at different complexity levels (high, medium, and low complexity). CAMI_low consists of one simulated Illumina HiSeq data, with size 15 Gbp and a total of 40 genomes and 20 circular elements. CAMI_medium includes two samples with a total size of 40 Gbp (132 genomes and 100 circular elements). CAMI_high is a simulated time series benchmark dataset with five samples of size 75 Gbp (596 genomes and 478 circular elements). All the samples in the three datasets are paired-end 150-bp Illumina reads and are available at CAMI web site (Sczyrba et al., 2017). CAMI datasets have been studied by several binning tools (i.e., CONCOCT (Alneberg et al., 2014), MyCC (Lin & Liao, 2016), COCACOLA (Lu et al., 2017), BinSanity (Graham, Heidelberg & Tully, 2017), MaxBin2 (Wu, Simmons & Singer, 2016), and MetaBat2 (Kang et al., 2019)). In their last report MetaBAT2 shows better performance that the other binning tools in all CAMI experiments. Therefore, we use MetaBAT2 to compare our results.

We downloaded the three synthetic datasets from the CAMI website and studied each individually. For the three CAMI experiments, we configured DATMA with default parameters. It starts by removing low-quality reads (quality Q < 30 and length <60 bp). Then, the remaining sequences were merged using the FLASH2 (Magoč & Salzberg, 2011) and forced to combine the reads that were not merged (only for binning purposes) using extra “NNN” because they were too large (see methods section). Then, the 16S rRNA ribosomal sequences were separated using BWA (Li & Durbin, 2009) to map the reads against the Rfam database (Griffiths-Jones et al., 2003). The remaining reads were binned with CLAME using b=70bp and iterating with b=50bp and b=30bp. We set DATMA to report only bins with more than 100.000 reads and selected SPAdes (Nurk et al., 2013) as the assembler tool. We used CheckM results, reported by DATMA, to assess the genome completeness and compare those results against the MetaBAT2 report. For all the experiments, we set the number of threads to twenty and configured DATMA to process blocks of 20 million reads. The complete configuration file, for each dataset, is available in DATMA’s GitHub.

**Figure 2:**
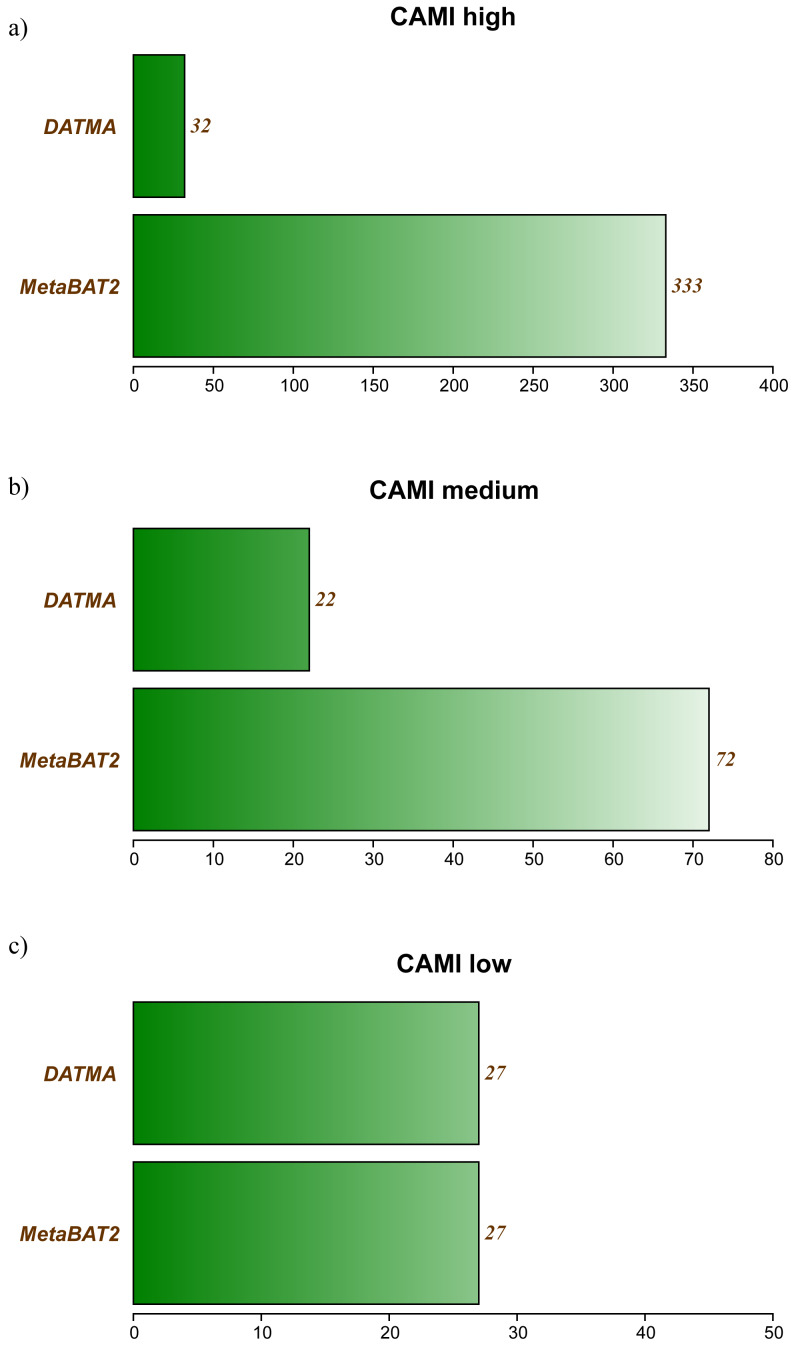
DATMA results for CAMI Low and Medium Complexity datasets. (A) CAMI high. (B) CAMI medium. (C) CAMI low.

Tables S2, S3 and S3 in Additional File 1, contains the full report generated by CheckM for the three CAMI datasets. Figure 2 summarizes the best bins (completeness >40% and low contamination) from each experiment. In the CAMI-low Complexity dataset, DATMA recovers 27 of 40 genomes present in the sample, with completeness higher than 60%. In the CAMI-medium experiment, it reports 28/132 genomes, most of them with completeness higher than 60%. In the CAMI_high dataset, DATMA reports 33/596 genomes, all of them with completeness higher than 40%. Figure 2 also compares our results against the MetaBAT2 report. It shows that MetaBAT2 has better performance than DATMA in all the experiments; however, it is essential to notice that DATMA uses the raw reads, while MetaBAT2 uses the golden bins (a set of well-defined assembly contigs provides by CAMI). These results suggest that DATMA can recover the predominant genomes from a complex metagenome of a microbial community. However, they also indicate the limitation of our tool to recover species in less abundance into the metagenome (only the most abundant were reported) or separate close-taxonomic genomes (high contamination level is present in some bins).

### Brocadia caroliniensis metagenome

We used a metagenome recovered from a full-scale glycerol-fed nitritation-denitritation separate centrate treatment process (NCBI project PRJNA228949). The original paper (Park et al., 2017) reports that 2,448,982 reads were manually analyzed to generate 209 contigs (with size >500 bp) that integrate the draft genome for *Brocadia caroliniensis* species. We downloaded the raw reads and analyzed them with DATMA. It was executed with default parameters to remove low-quality bases and reads that were too short (Q<30 and length <70 bp). The 1,860,653 leftover reads were aligned against the Rfam database (Griffiths-Jones et al., 2003) to remove 16S rRNA gene sequences. After removing 12,754 reads, DATMA called CLAME (Benavides et al., 2018) with 1,847,899 sequences using b=70bp, as the number of bases alignment parameter. The bins with more than 2000 reads were assembled with SPAdes (Nurk et al., 2013).

Table 1 summarizes the number of bins generated, the assembly metrics, the total ORFs detected, the completeness-contamination of the bins, and the computational time used by DATMA. It also contrasts these results against the report produced by MetaWRAP (Uritskiy, DiRuggiero & Taylor, 2018) and SqueezeMeta (Tamames & Puente-Sánchez, 2019) frameworks. MetaWRAP completeness of the Brocadia genome is higher than the obtained by DATMA; but, DATMA obtains a better N50. SqueezeMeta annotated most reads as Brocadicae family, but it generated a larger number of contigs than the other frameworks. DATMA was the fastest tool.

We configured DATMA to use Quast tool (Gurevich et al., 2013) with a reference. It allowed evaluating the coverage and depth of the bins generated by our pipeline. Table 2 shows that DATMA assembly covers about 97% of the Brocadia genome, and only six contigs did not align with precision to the reference sequence. DATMA covered more of the genome than the other tools, but it presented more unaligned contigs than MetaWRAP. DATMA had similar results than the manual process of the original paper but it was automatic and faster than the other tools.

### San Fernando biosolid metagenome

We used DATMA to study the biosolid metagenome produced by the San Fernando wastewater plant located in Medellin-Colombia. Two biosolid samples (each about 0.5 kg) were collected and transferred to the laboratory in refrigeration. The DNA extraction was done using PowerMax^®^ Soil DNA Isolation Kit supplied by MOBIO Corporation (Diagnostics Products MP Biomedicals, 2019). The samples were then sequenced using ROCHE’s 454 Titanium technology in 3/4 PTP at the Centro Nacional de Secuenciación Genómica-CNSG, Universidad de Antioquia, Medellin, Colombia. A total of 6,206,317 reads were analyzed. A study of the microbial diversity, as well as the methanogenesis pathway of this metagenome, is presented in Bedoya et al. (2019).

**Table 1:** Analysis report for the Brocadia experiment.

**Tool**	**TotalBins**	**TotalContigsper bin**	**Contigs’ metrics(Report from Quast tool)**				**Recovered genome(Report from CheckM tool)**			**Time(m)**
			Largest (bp)	N50 (bp)	Genome (Mbp)	ORFS	Complete -ness (%)	Contami -nation (%)	Lineage	
**DATMA**	2	677	88819	18421	3.96	4330	93.96	10.05	Brocadiaceae	60
		1382	13527	2456	2.37	3656	47.95	1.78	Brocadiaceae	
**MetaWRAP**	2	607	58497	9402	3.67	4273	96.08	5.00	Brocadiaceae	135
		374	29910	10268	2.81	4015	77.30	1.75	Brocadiaceae	
**Squeeze-Meta**	(NA*)	10345	3264	519	4.13	10283	89.47	111.28	Brocadiaceae	85
		12753	3420	360	4.21	12607	74.76	100.00	Bacteroidetes	
		12698	4314	342	4.14	11916	65.33	84.78	Proteobacteria	

**Notes.**

* We manually selected the contigs from the annotation report.

**Table 2:** Coverage report on the Brocadia genome using the contigs from each framework.

	**NumContigs**	**NG50**	**MisassembledContigs**	**UnalignedContigs**	**GenomeFraction (%)**	**DuplicationRatio**
DATMA	677	19785	53	6	97.2	1.0
MetaWRAP	607	9191	73	2	96.4	1.0
SqueezeMeta	10345	579	5	1251	30.4	1.0

DATMA was executed with default parameters to remove low-quality sequences (Q<30 and length <70 bases) and 5,668,260 reads were left. These reads were aligned against the Rfam database (Griffiths-Jones et al., 2003) to identify 16S rRNA ribosomal sequences. A total of 53,557 reads were detected and separated by DATMA. The 5,614,703 leftover sequences were binned with CLAME using default parameters but reporting bins with more than 5000 reads. We selected SPAdes (Nurk et al., 2013) as the assembler tool. We compared DATMA’s results and performance against MetaWRAP (Uritskiy, DiRuggiero & Taylor, 2018) and SqueezeMeta (Tamames & Puente-Sánchez, 2019) frameworks. Moreover, we used MG-RAST server (Wilke et al., 2016) to analyze this metagenome. We set the number of threads to four for all pipelines. We used Squeezeemeta in co-assembly mode using two samples collected in different seasons of the year. However, it generated a No-Consensus output in the merge stage. Therefore, we configured it in sequential mode and executed it with the complete biosolid dataset.

**Figure 3:**
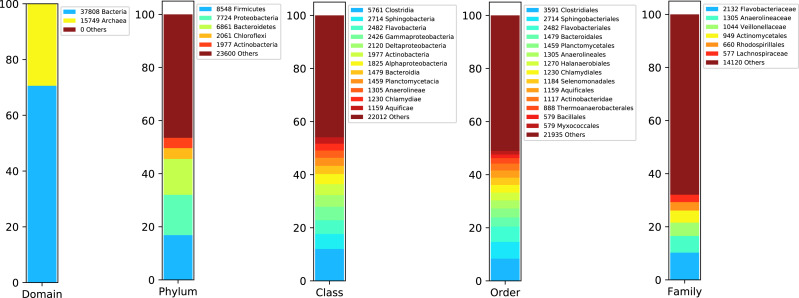
Taxonomic report for the 16S rRNA ribosomal sequences from Biosolid metagenome.

We started rating the species abundance in the metagenome using the 16S rRNA sequences reported by DATMA. Figure 3 shows the taxonomic annotation generated by the RDP Classifier tool (Wang et al., 2007) with the ribosomal reads. It indicates that Bacteria, with 70% of the reads, is the primary domain. Within this domain, Firmicutes, Proteobacteria, Bacteroidetes, and Chloroflexi are the main phyla (with 16%, 14%, 13%, and 3% respectively). Each of them contains several families, except for Chloroflexi, in which case Anaerolineaceae is the dominant family (it corresponds to the 3% of all metagenome).

Table 3 summarizes the following results obtained by DATMA, MetaWRAP (Uritskiy, DiRuggiero & Taylor, 2018) and SqueezeMeta (Tamames & Puente-Sánchez, 2019): number of contigs, their metrics, the ORF detected, the number of genomes reported and the quality according to the CheckM tool (Parks et al., 2015). We have included the report generated by the MG-RAST server (Wilke et al., 2016). MetaWRAP reports eight genomes with completeness higher than 80% and a contamination level less than 7%. However, most of them cannot be assigned with precision into a clade family, and most importantly, no bin was annotated into Anaerolineaceae family (see Fig. S4, in Additional File 3). SqueezeMeta shows that Proteobacteria is the dominant phylum, but no bins belonging to Chlorofexi were reported. MG-RAST indicates that most of the reads are classified as Pseudomonadaceae and Anaerolineaceae families, but because we submitted the data as a private project, no additional information could be collected. We observed that DATMA is the only tool which reports two primary bins annotated in the Anaerolineaceae family. Moreover, it was the fastest tool.

**Table 3:** Analysis report for the Biosolid metagenome.

	**TotalBins**	**TotalContigsper bin**	**Contigs’ metrics(Report from Quast tool)**				**Recovered genome(Report from CheckM tool)**			**Time(m)**
			**Largest(bp)**	**N50(bp)**	**Genome(Mbp)**	**ORFs**	**Complete-ness(%)**	**Contami-nation(%)**	**Lineage**	
**DATMA**	2	1292	12707	2912	2.85	3786	54.81	101.18 (72.60%)a	Chloroflexi-Anaerolineaceae	125
		647	37610	5380	2.10	2529	70.69	49.34 (97.75%)a	Chloroflexi-Anaerolineaceae	
**MetaWRAP**	8	495	87496	17399	4.92	4266	94.59	4.73	Bacteria	485
		157	82800	18931	2.10	2110	89.03	1.69	Bacteria	
		218	60788	20930	2.72	2954	88.70	3.22	Proteobacteria	
		463	34164	7341	2.57	3031	87.16	1.32	Bacteria	
		731	22922	4571	2.78	3293	85.49	3.01	Actinobacteria	
		994	23123	4103	3.58	4481	84.98	6.70	Proteobacteria-Pseudomonas	
		420	26523	6363	2.17	2969	83.09	1.11	Gammaproteobacteria	
		754	19037	5384	3.33	3944	82.64	3.61	Proteobacteria-Pseudomonadaceae	
**Squeeze-Meta**	NA (b)	204323	11961	528	93.69	46730	100	2844	Proteobacteria	626
		49288	5376	579	24.30	49227	95.83	1258	Firmicutes	
		47728	5055	519	21.66	46730	100	675	Actinobacteria	
		41526	9084	585	20.69	41342	100	659	Bacteroidetes	
**MG-Rast**	NA (b)	114806 (c)	NA	NA	NA	NA	NA	NA	Proteobacteria-Pseudomonadaceae	1 week
		95148 (c)							Chloroflexi-Anaerolineaceae	

**Notes.**

a Strain-heterogeneity index.

b We manually selected the contigs from the annotation report.

c The values correspond to number of reads.

According to MIMAG standards (Bowers et al., 2017) to report a genome, Bin0 with 1292 contigs and Bin1 with 647 contigs have suitable results to propose a draft genome. However, the contamination level indicated in Bin0 is too high to propose a draft genome. We focus our study on Bin 1.

**Figure 4:**
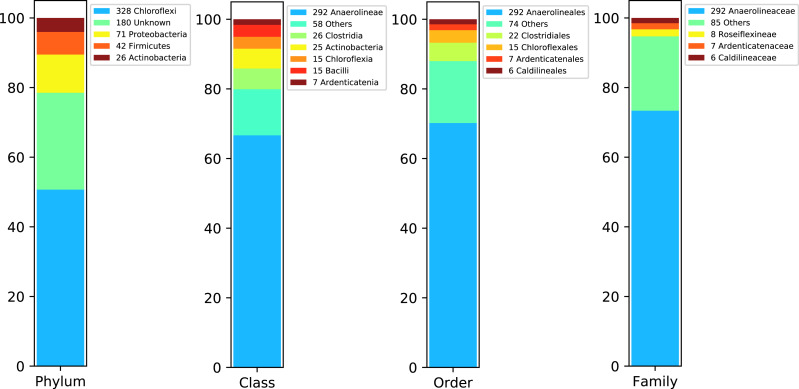
Taxonomic report for the Bin 1 from Biosolid metagenome using DATMA.

Figure 4 shows DATMA’s annotation report using the Kaiju tool (Menzel, Ng & Krogh, 2016) for Bin 1. It indicates that most of the contigs were annotated into the Chloroflexi phylum and Anaerolineaceae family. Moreover, the relation between the number of ORFs and the genome estimation (1 ORF per Kbp) agrees with the relation reported for similar species from this family (i.e., *Pelolinea submarina* with 3131 ORFs, 3.5 Mbp and a relation of 0.89 ORFs/Kbp and *Leptolinea tardivitalis* with 3301 ORFs, 3.69 Mbp and a relation of 0.90 ORFs/Kbps). We mapped the 53,557 reads into the 647 contigs and computed the assembly depth. It presents an average of 18.94 reads per position.

**Figure 5:**
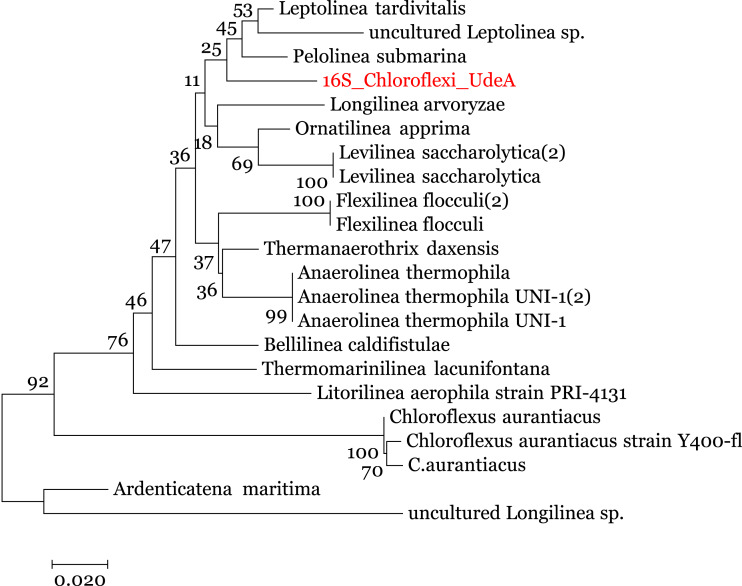
Phylogenetic tree for the 16S rRNA ribosomal gene (16S_Chloroflexi_UdeA). The values in the branches indicate the percentage of replicate trees in which the associated taxa clustered together in the bootstrap test.

We enriched the contigs with the 16S rRNA sequences removed in the 16S-identification stage. We manually selected the reads annotated within the Anaerolineaceae family and assembled them using the SPAdes (Nurk et al., 2013) to obtain the complete 16S rRNA gene. Analysis of this 16S rRNA ribosomal gene, using BLAST (against the local NT) indicated that the 16S rRNA gene is related to *Anaerolinea thermophila UNI-1 DNA* species (NCBI Accession AP012029). To improve the taxonomic annotation, we used MEGA 7.0 (Kumar, Stecher & Tamura, 2016) to build a phylogenetic tree using the 16S rRNA sequences and the Ribosomal data project database (Cole et al., 2014). The evolutionary tree, in Fig. 5, was inferred by using the Maximum Likelihood method with the Jukes-Cantor model (Kumar, Stecher & Tamura, 2016) and the process described by Brumm et al. (2015). It indicates that the recovered reads are close to the family Anaerolineaceae and are related to the genus Pelolinea and Leptolinea.

Using the set of standards for the minimum information regarding a metagenome-assembled genome (MIMAG) proposed by Bowers et al. (2017) and the previous results, we can use the contigs from Bin 1 to describe a Low-quality draft genome that belongs to the family Anaerolineaceae, which is closely related to the genus Pelolinea and Leptolinea. We called this draft genome Anaerolineaceae_UdeA_SF1. We submitted the draft genome (assembled contigs and respective reads) to the NCBI database (Bioproject PRJNA529916).

### Computational performance

To illustrate the computational performance of DATMA we executed the experiments within two different scenarios: (i) single mode, using only the Master machine, and (ii) distributed mode, using the Master machine with multiple workers like a grid of computers. We simulated the grid of computers using tree servers (Master, Worker1, and Worker2) connected via a secure shell connection. Table S5, in Additional File 1, illustrates the computer specifications of each server. To simulate a more significant number of workers, like a bigger grid of computing, we allow for several tasks to run on the same computer. Applications were configured to use four threads on all the experiments.

**Figure 6:**
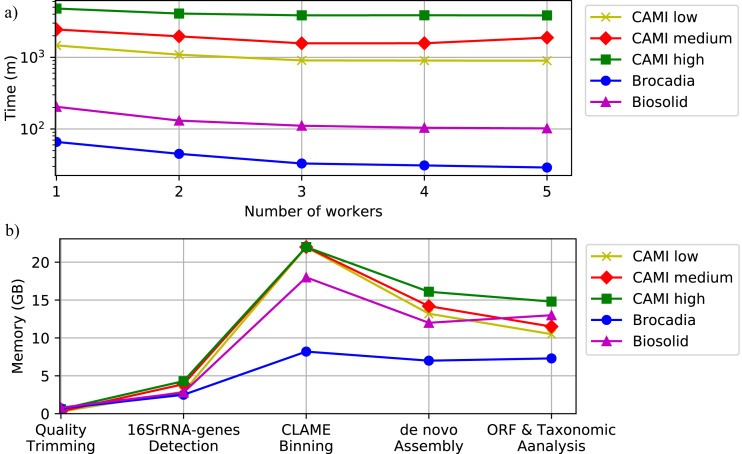
Computational performance of DATMA. (A) Computational time of DATMA for all datasets using several workers. (B) Memory performance of DATMA.

Figure 6 and Table S6, in Additional File 1, show the execution time for all the datasets using several scenarios. It shows that computational time decreases as the number of workers increase. Figure 6 also illustrates the memory performance of DATMA. It reports a peak in the binning stage, but it then decreases when DATMA distributes the next tasks into the available computing resources. Because we used blocks of the same size (20 million reads) to bin the raw reads of the CAMI datasets, the peak of memory is the same for all the experiments. It can be reduced by modifying the blocks size parameter, in the configuration file.

## Discussion

DATMA is designed to extract Bacteria genomes, addressing two typical challenges of metagenomic projects: (i) metagenomics assembly, a complex task due to the mix of reads from several species, and (ii) the computational time required to analyze the massive amount of data recovered with NGS technologies.

Since binning the reads before de novo assembly increases the quality of downstream assembly and analysis, we designed DATMA around CLAME (Benavides et al., 2018). This binning tool improves the contigs’ quality by grouping reads from a single molecule into the same bin, which also reduces the assembly time. DATMA integrates CLAME with other NGS tools. The current version of DATMA includes the stages that we consider to be the main features of a full metagenomics analytical workflow; however, the modular structure used for developing DATMA allows that new tools can be included in future versions.

DATMA also addresses the computational constraints of a single computer. It takes advantage of CLAME’s binning and uses COMPSs (Badia et al., 2015) to automatically distribute the processing of the bins into a distributed computer structure. Even though exploiting parallelism is a complex task, by using COMPSs, DATMA can exploit parallelism from high-level abstraction despite the dataset complexity or the heterogeneousness of the computer resources. Moreover, DATMA showed to be useful to recover and annotate genomes from metagenomic datasets faster as the number of computers increase. Although a direct matching of DATMA execution times with other frameworks is not feasible due to the differences in the structure, number of stages, different software tools used, and design of each framework, DATMA was still faster than the other employed pipelines in studying the experimental metagenomes presented in this paper and produced similar results.

We show DATMA’s functionality using the CAMI challenge datasets, currently, the most popular benchmark datasets for metagenomic binning and assembly testing. For the three CAMI dataset (low, medium, and high), DATMA effectively grouped reads with low contamination and recovered most genomes present in each sample. Since DATMA used the raw reads instead of the GoldStandardAssembly contigs, as it is done in the MetaBAT2 paper, DATMA cannot match its performance. However, to this date, we did not find an assembly tool for metagenomics that can produce a gold assembly, so for real metagenomes those binning results, cannot be matched. Moreover, the assembly of all the datasets is a demanding computing task that can exceed the computing capacities of most servers that are not supercomputers. Since DATMA creating bins of reads previously to the assembly task, the assembler can assemble the complete metagenome in parts employing less computing resources. Since DATMA is stricter than the other tools creating the bins, a genome can sometimes be split among different bins, producing a trade-off between purity and completeness.

Although a metagenomic read dataset contains a mixture of sequences from several species, in some cases there is an organism with enough reads to extract the genome from it. The Brocadia-metagenome experiment is an example of this scenario. In this metagenome, CLAME binned most of the reads from the predominant species into a single bin. Then, DATMA assembled the reads of the bin generating similar results to the original paper; however, they needed several manual steps to get the final results. We measured the genome completeness using single-copy universal genes and found that DATMA recovered most of the genome. While the other frameworks also recovered a similar proportion of the genome, DATMA was the fastest tool.

On a complex metagenome, like the San Fernando biosolid metagenome, DATMA generated the bacterial profile of the dominant microorganisms. It indicates that Proteobacteria is the predominant phylum; however, there are several families in this phylum. Chloroflexy is not the dominant phylum, but it looks like the predominant species. CLAME was the only binning tool that grouped most of the reads of this predominant genome into a bin, then DATMA used SPAdes (Nurk et al., 2013) to assemble and BLAST (Altschul et al., 1990) to annotate the bin as an Anaerolineaceae family. The taxonomic assignation was corroborated using the 16S rRNA gene phylogenetic analysis. It showed that DATMA extracted most reads from a novel taxon that belongs to the family Anaerolineaceae of the class Anaerolineae, closely related to the genus Pelolinea and Leptolinea, from a complex metagenome. Because this Anaerolineaceae is very likely a new family, MetaWRAP was not able to recover it, since this framework relies heavily on CheckM to create the groups and it works based on known species.

## Conclusions

We have developed DATMA, an automatic metagenomic framework, which integrates the CLAME binning tool with other state-of-the-art omics tools and allows a full analysis of metagenomic datasets. Based on the binning strategy and Master-Workers model, DATMA processes metagenomes using distributed computing, providing quality assembly and faster annotation, and in many cases better than other similar frameworks. Because DATMA does not rely on known species to produce the bins, it is better than other frameworks for new species or families. DATMA also provides bins with low contamination (based on universal single-copy markers) because it is very strict in creating them. We showed DATMA functionality analyzing a very complex metagenome and how it automatically extracts an almost complete genome from its predominant species.

##  Supplemental Information

10.7717/peerj.9762/supp-1 Supplemental Information 1 Detail CLAME binning process using MAD statistic metric

10.7717/peerj.9762/supp-2 Supplemental Information 2 Supplementary figures

## Additional Information and Declarations

### Data Availability

The following information was supplied regarding data availability: All the data generated or analyzed during this study are available in DATMA’s GitHub: https://github.com/andvides/DATMA.
