# Supplementary material for: DATMA: Distributed AuTomatic Metagenomic Assembly and annotation framework
Source: PeerJ. 2020 Sep 3;8:e9762. doi: 10.7717/peerj.9762 (PMC7474881; doi:10.7717/peerj.9762)
Supplement: Supplemental Information 1 [file peerj-08-9762-s001.pdf]

Detail CLAME binning process using MAD statistic metric

S.1 Introduction

CLAME bins DNA sequences from a metagenome using overlapping read detection. It reports the relation found by each read as an adjacency list in which the first column represents the node (one read) and the second, the edges (the sequences that align in at least b bases with this read) (Figure S.1 (a)). Therefore, the adjacency list is a graph representation of the alignments of the reads. This graph is examined to report each subgraph as the bins (Figure S.1 (b)). To be analyzed, it is necessary to set thresholds, on the number of edges, to separate subgraphs that connect two species closely related that are in different concentration. In the original CLAME paper, the authors provide a histogram of the number of edges per read, to set the thresholds and separate subgraphs. To configure this parameter automatically, DATMA uses the median absolute deviation (MAD). It does it by discarding the outliers (Figure S.1 (c)). DATMA incorporates the calculation of the threshold using MAD as a C programming function inside of CLAME's code. CLAME's version that includes MAD process is available on DATMA GitHub.

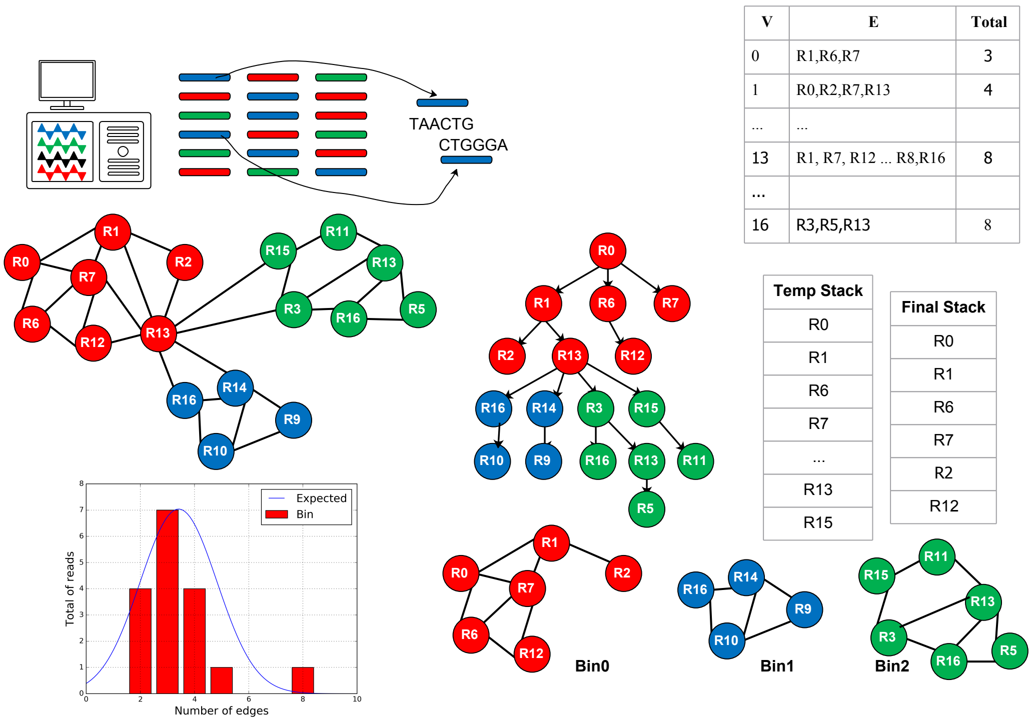

Figure S.1: CLAME methodology. a)The metagenome contains reads from different genomes (red, blue, and green blocks); CLAME aligns each read against all the reads. b)An adjacency list represents a graph $G = (V, E)$, where each vertex v in V denotes a read and each edge e in E indicates that two reads align in at least b bases. The bins are generated by traversing the graph and reporting each subgraph into a temporal stack (e.g., R0, R1, R6 R13, R5). Reads that belong to a shared region can connect the subgroups (i.e., R13 from red group aligns with the R3, and R15 from the green group). c)These connections usually make that the number-of-edges histogram departs from a normal like form. Edges analysis removes sequences with extreme values (i.e., R13), and report the final bin (e.g., R0, R1, R6, R7, R2, R12). The graph is traversed several times until all the reads are binned.

S.1.1 The median absolute deviation (MAD) scale estimator

The MAD is a robust nonparametric spread estimator. MAD uses the median instead of mean to estimate the amount of data dispersion. The median, like the mean, is a measure of the central tendency of a random variable, but, as opposed to the mean, it is very insensitive to outliers. The MAD is defined as:

$$MAD = median(|x_i - median(x_i)|) \quad \text{(Eq. S.1)}$$

For a normal distribution, the MAD can be used as a consistent estimator of the population standard deviation as:

$$\sigma' = b * MAD \quad \text{(Eq. S.2)}$$

where b is a constant scale factor; for normally distributed data b=*1.4826*.

This reworked form of σ' allows flagging outliers by considering distances from the median (M). The decision criterion (for the value of 3) becomes:

$$M - 3 * \sigma' < x_i < M + 3 * \sigma' \quad \text{(Eq. S.3)}$$

S.1.2 Outliers relation with the maximal and minimal number of edges by node

Since the distribution on the number of edges per node departures from a normal, because of the noise produced by the similarity of regions of the genome with other genomes or repetitive zones, we can use MAD (according to the Eq. S.2) to compute the population standard deviation of the number of edges per read in the bins and remove outliers. We use the MAD to mark sequences out of the three standard deviations as outliers, (according to the Eq. S.3), and separate them. After separating outliers, it is common that the bins number of edges distribution becomes normal.

Furthermore, from the characteristics of a normal distribution and because it is not possible to have nodes with the number of edges less than zero, we can define the parameter p (in Eq. S.4) as the measure of normality for the bin.

$$p = \frac{3 * \sigma''}{\mu''} \qquad \text{(Eq. S.4)}$$

where the μ'' and σ'' are the mean and standard deviation of the bin, after the outliers removing process.

Since approximately 95% of points taken from a normal distribution should be not more than three standard deviations from the mean (see FigureS.2); a p-value close to one indicates that the bin has a near normal distribution. Therefore the p-parameter allows assessing the outlier removing process and iterate removing new outliers on the bin to reach a p-value close to 1.0. We define a tolerance value (the *tol*-parameter, with a default value of 0.5) to stop the process, it can also finish when the bin is too small to be reported. Table **??** illustrates the Edge analysis process for the example in Figure S.1. The experimental section exhibits MAD convenience to remove outliers and produce "pure" bins (in which most of the reads are from the same molecule).

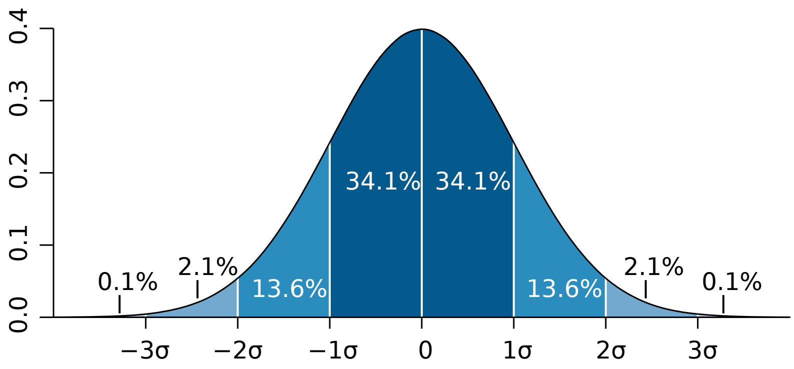

Figure S.2: Probability density function for a normal distribution

**Read**	x_i	M_j	$abs(x_i - M_j)$	M_i	**MAD**	$(xi - Mj)/MAD > \|\pm 3\|$	**outlier**
R0	3	3	0	1	1.4826	0	NO
R1	4		1			0.67	NO
R2	2		1			0.67	NO
R6	3		0			0	NO
R7	5		2			1.35	NO
R12	3		0			0	NO
**R13**	**8**		**5**			**3.37**	**YES**
R9	2		1			0.67	NO
R10	3		0			0	NO
R14	4		1			0.67	NO
R16	3		0			0	NO
R3	4		1			0.67	NO
R5	2		1			0.67	NO
R11	2		1			0.67	NO
R13	4		1			0.67	NO
R15	3		0			0	NO
R17	3		0			0	NO
**mean**	3.41	3.33					
**std**	1.46	1.03					
**p=3std/mean**	1.28	0.92					

Table S.1: Edge analysis example using MAD to detect outliers. Let us consider the adjacency list X_i, which indicates the number of edges per node. It has an original $mean = 3.4$, a standard deviation $std = 1.46$, and a median $M_j = 3$. The p-value = 1.28 indicates a non-normal distribution. Edge analysis stage subtracts the median from each observation to get the new median $M_i = 1$. It will be multiplied by 1.4826 to find a $MAD = 1.48$ (Eq. S.1 and Eq. S.2). MAD reports that the read R13, with total edges, equal 8, is an outlier (according to the Eq. S.3) and removes it. Removing this point the new statistic parameters are: $mean = 3.3$, $std = 1.03$ and p-value = 0.93. The new p-value is close to one, which indicates a near-normal distribution and stops the Edge analysis process
