# Supplementary material for: DATMA: Distributed AuTomatic Metagenomic Assembly and annotation framework
Source: PeerJ. 2020 Sep 3;8:e9762. doi: 10.7717/peerj.9762 (PMC7474881; doi:10.7717/peerj.9762)
Supplement: Supplemental Information 2 [file peerj-08-9762-s002.pdf]

Supplementary figures

a) **Reads report**

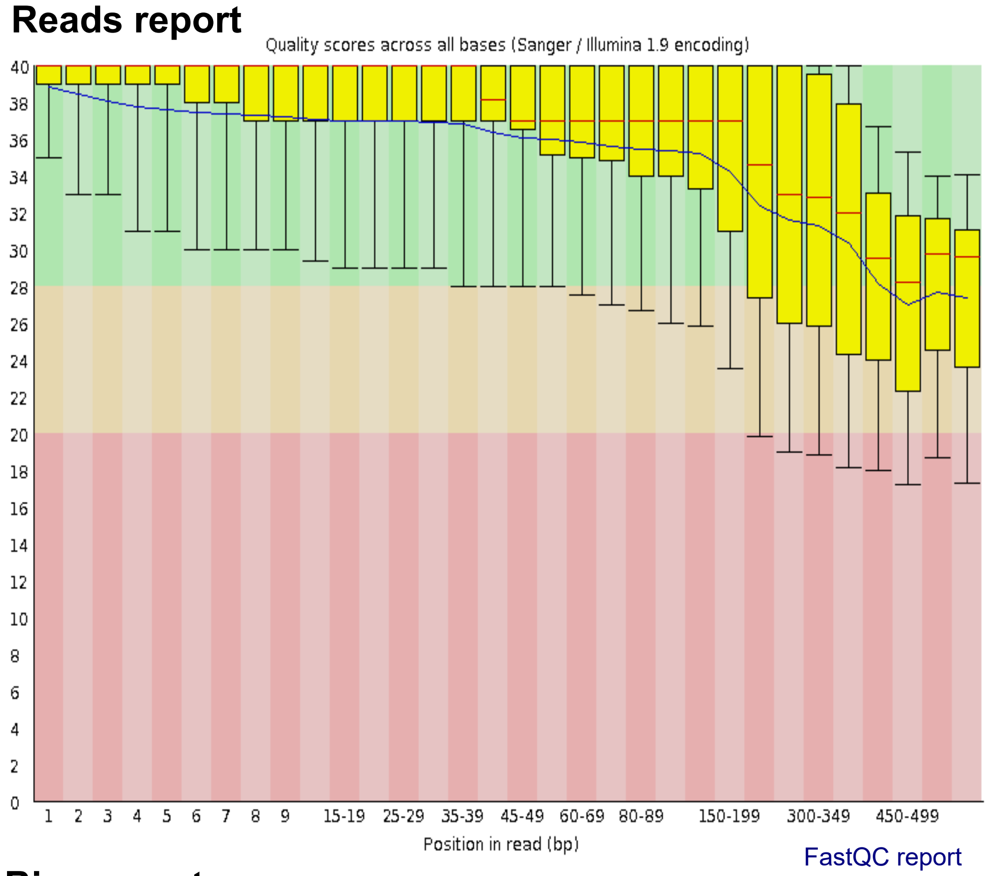

FastQC report

b) **Bins report**

Bin	Size(reads)	bp	Contigs	Genome	ORFS	bp	Link
all_16S	9957	3576767	NA	NA	NA	NA	NA
Bin0	266869	81569006	131	3243019	3124	943731	fullLink
Bin1	335701	135467115	151	4198482	3985	1259949	fullLink
Bin2	1271	531435	2	4217	7	1240	fullLink
Bin3	1252	496908	1	14521	15	4380	fullLink

c) **Assembly report**

Bin Id	Marker lineage	UID	genomes	markers	marker sets	0	1	2	3	4	5+	Completeness	Contamination	Strain heterogeneity
Bin1	Mycobacterium	UID1816	100	690	300	15	674	1	0	0	0	97.54	0.33	0.00
Bin0	Brucella	UID3486	87	1402	225	25	1372	5	0	0	0	97.24	0.28	20.00
Bin4	root	UID1	5656	56	24	56	0	0	0	0	0	0.00	0.00	0.00
Bin3	root	UID1	5656	56	24	56	0	0	0	0	0	0.00	0.00	0.00
Bin2	root	UID1	5656	56	24	56	0	0	0	0	0	0.00	0.00	0.00

Figure S.1: DATMA output. a)Reads Quality, b) CLAME report and c)Assembly metrics for every bin

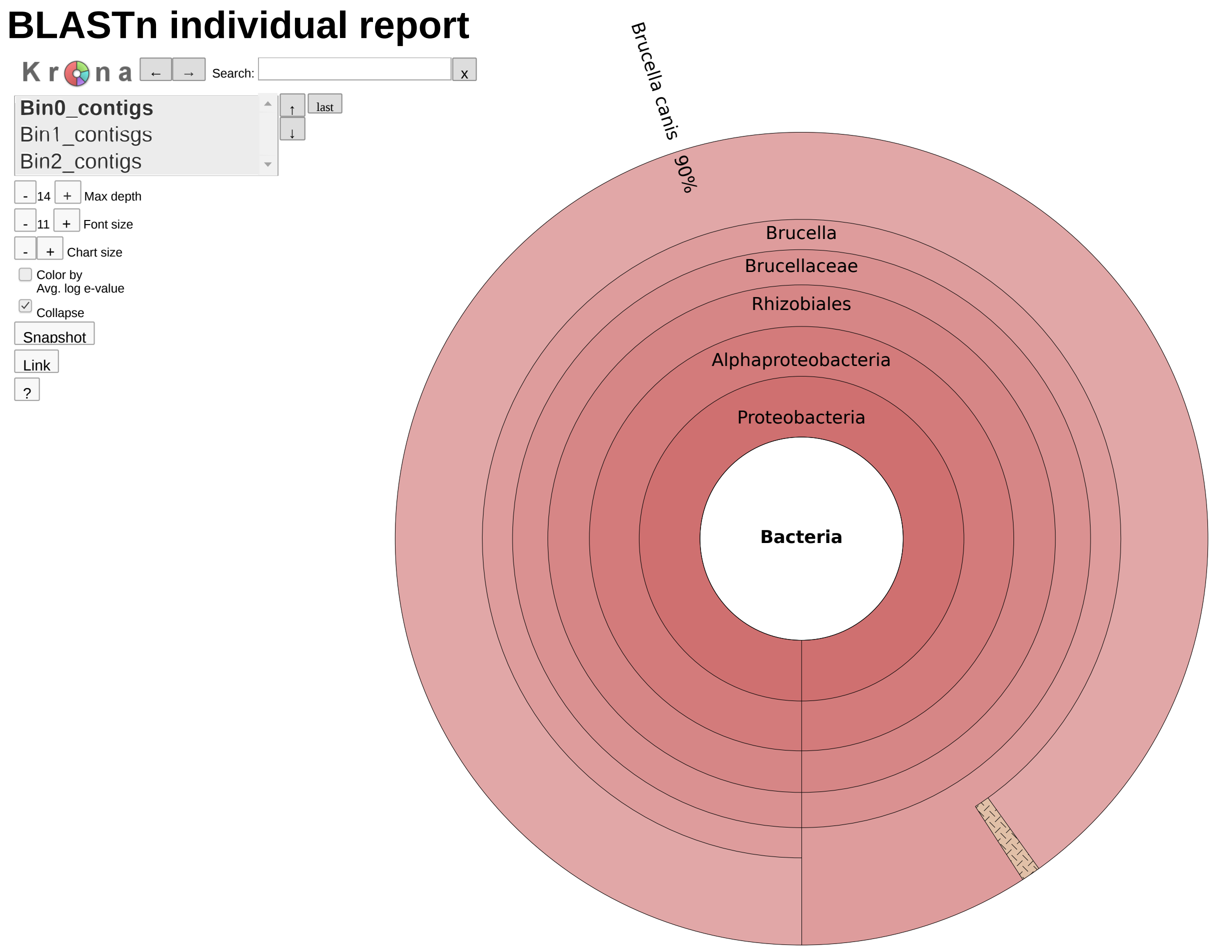

Figure S.2: DATMA output. Taxonomic annotation for each bin

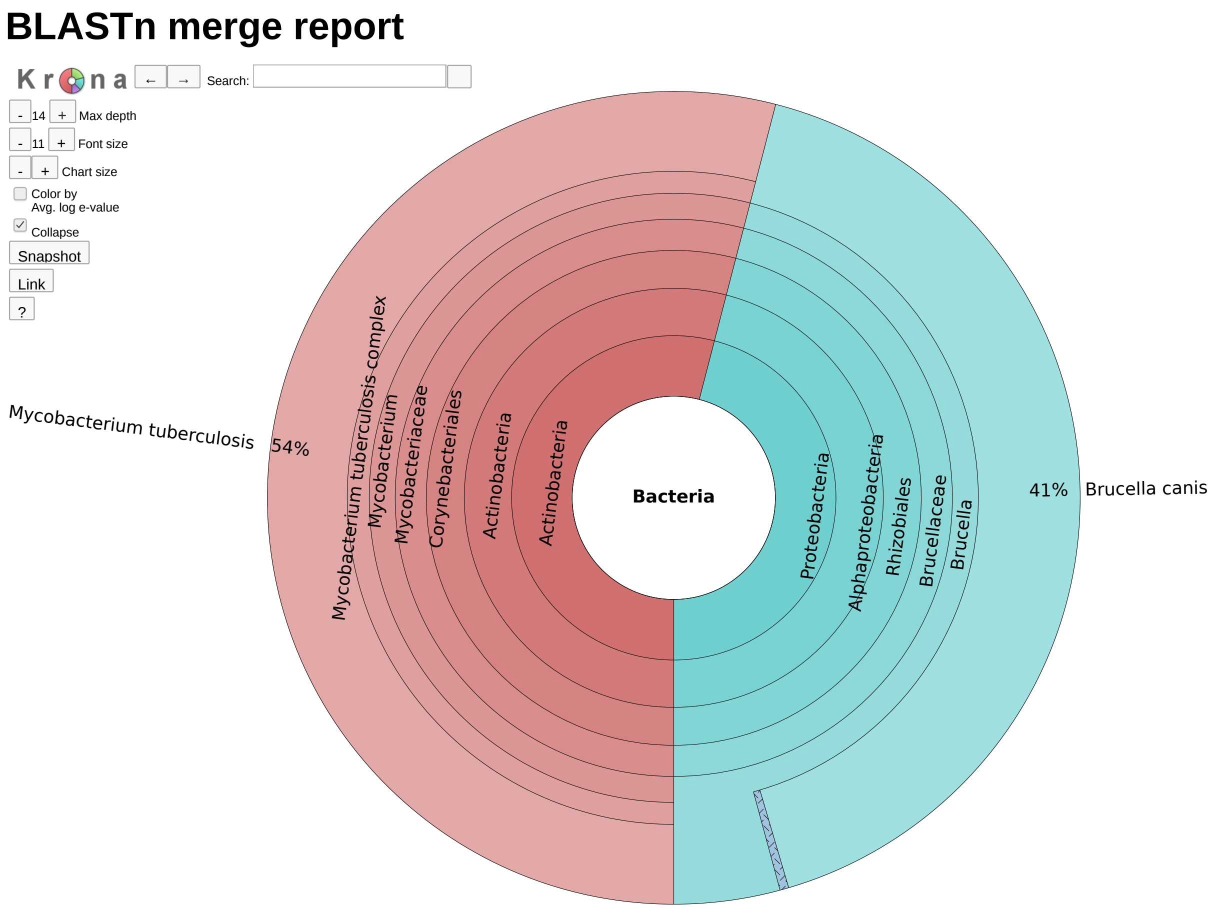

Figure S.3: DATMA output. Taxonomic annotation for all bins

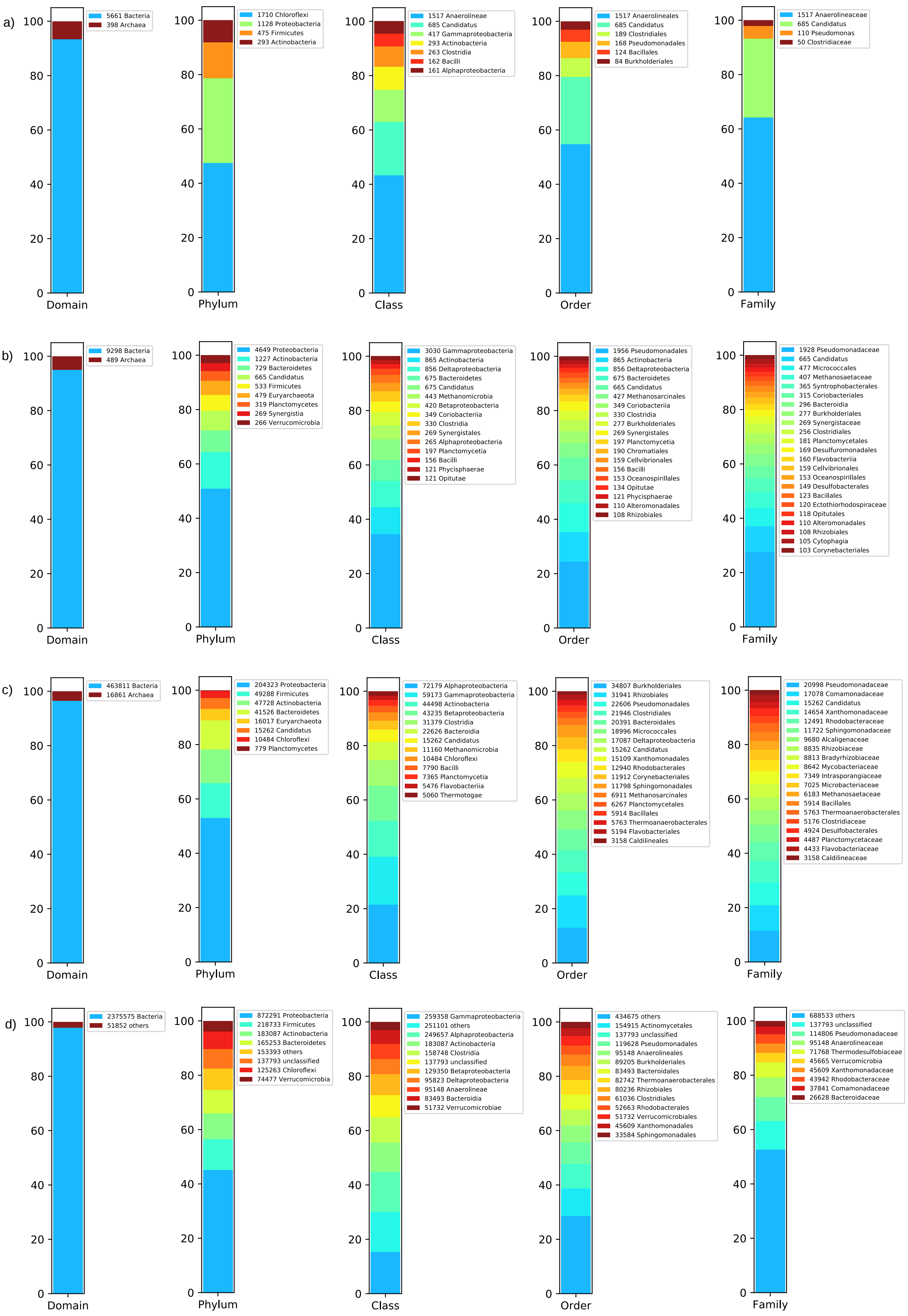

Figure S.4: Taxonomic report for the biosolid metagenome using: a)DATMA, b)MetaWRAP, c)SqueezeMeta, and d)MG-RAST
